# Novel Privacy Preserving Non-Invasive Sensing-Based Diagnoses of Pneumonia Disease Leveraging Deep Network Model

**DOI:** 10.3390/s22020461

**Published:** 2022-01-08

**Authors:** Mujeeb Ur Rehman, Arslan Shafique, Kashif Hesham Khan, Sohail Khalid, Abdullah Alhumaidi Alotaibi, Turke Althobaiti, Naeem Ramzan, Jawad Ahmad, Syed Aziz Shah, Qammer H. Abbasi

**Affiliations:** 1 Department of Electrical Engineering, Riphah International University, Islamabad 46000, Pakistan; Arslan.shafique@riphah.edu.pk (A.S.); s.khalid@riphah.edu.pk (S.K.); 2 Department of Computer Sciences, RMIT University, Melbourne 3000, Australia; kashifhesham.khan@rmit.edu.au; 3 Department of Science and Technology, College of Ranyah, Taif University, P.O. Box 11099, Taif 21944, Saudi Arabia; a.alhumaidi@tu.edu.sa; 4 Department of Computer Science, Faculty of Science, Northern Border University, Arar 91431, Saudi Arabia; turke.althobaiti@nbu.edu.sa; 5 Remote Sensing Unit, Northern Border University, Arar 91431, Saudi Arabia; 6 School of Computing, Engineering and Physical Sciences, University of the West of Scotland, Paisely PA1 2BE, UK; naeem.ramzan@uws.ac.uk; 7 School of Computing, Edinburgh Napier University, Edinburgh EH10 5DT, UK; J.Ahmad@napier.ac.uk; 8 Research Centre for Intelligent Healthcare, Coventry University, Coventry CV1 5FB, UK; syed.shah@coventry.ac.uk; 9 James Watt School of Engineering, University of Glasgow, Glasgow G12 8QQ, UK; qammer.abbasi@glasgow.ac.uk

**Keywords:** encryption, security, deep learning, machine learning, chaos

## Abstract

This article presents non-invasive sensing-based diagnoses of pneumonia disease, exploiting a deep learning model to make the technique non-invasive coupled with security preservation. Sensing and securing healthcare and medical images such as X-rays that can be used to diagnose viral diseases such as pneumonia is a challenging task for researchers. In the past few years, patients’ medical records have been shared using various wireless technologies. The wireless transmitted data are prone to attacks, resulting in the misuse of patients’ medical records. Therefore, it is important to secure medical data, which are in the form of images. The proposed work is divided into two sections: in the first section, primary data in the form of images are encrypted using the proposed technique based on chaos and convolution neural network. Furthermore, multiple chaotic maps are incorporated to create a random number generator, and the generated random sequence is used for pixel permutation and substitution. In the second part of the proposed work, a new technique for pneumonia diagnosis using deep learning, in which X-ray images are used as a dataset, is proposed. Several physiological features such as cough, fever, chest pain, flu, low energy, sweating, shaking, chills, shortness of breath, fatigue, loss of appetite, and headache and statistical features such as entropy, correlation, contrast dissimilarity, etc., are extracted from the X-ray images for the pneumonia diagnosis. Moreover, machine learning algorithms such as support vector machines, decision trees, random forests, and naive Bayes are also implemented for the proposed model and compared with the proposed CNN-based model. Furthermore, to improve the CNN-based proposed model, transfer learning and fine tuning are also incorporated. It is found that CNN performs better than other machine learning algorithms as the accuracy of the proposed work when using naive Bayes and CNN is 89% and 97%, respectively, which is also greater than the average accuracy of the existing schemes, which is 90%. Further, K-fold analysis and voting techniques are also incorporated to improve the accuracy of the proposed model. Different metrics such as entropy, correlation, contrast, and energy are used to gauge the performance of the proposed encryption technology, while precision, recall, F1 score, and support are used to evaluate the effectiveness of the proposed machine learning-based model for pneumonia diagnosis. The entropy and correlation of the proposed work are 7.999 and 0.0001, respectively, which reflects that the proposed encryption algorithm offers a higher security of the digital data. Moreover, a detailed comparison with the existing work is also made and reveals that both the proposed models work better than the existing work.

## 1. Introduction

Digital medical images such as X-ray images are becoming more crucial for identifying and treating disorders in contemporary hospitals and hence are attracting attention from the research community [1]. In general, such medical images may include confidential and sensitive information [2]. Disastrous accidents may arise if unauthorized access results in the theft of confidential information or the exploitation of these private images. For instance, a hacker may use unauthorized images or replace the original data with fabricated information or data to ruin a patient’s life. Furthermore, it is crucial to protect such medical images by means of some lightweight chaos-based encryption schemes. As a result, an unauthorized person cannot access the relevant database without the permission of the authorized party.

### 1.1. Data Security

Preserving images from unauthorized entities has become an increasingly important problem as multimedia technology continues to advance at an exponential rate. Compared to text data, images have distinct properties such as a large data capacity, high redundancy, and significant correlation between neighboring pixels, which make traditional text encryption algorithms inefficient when used on images. The development of acceptable image cryptosystems has been the focus of numerous studies, which have implemented a variety of techniques, including the Fourier transform [3,4], the wavelet transform [5], SCAN [6], chaos [7,8], and other techniques. Among the aforementioned methodologies, chaos-based image encryption is considered as one of the most efficient and effective encryption techniques due to a number of properties such as its high sensitivity to control parameters and initial values, pseudo random evolution, state ergodicity, and structure complexity [9].

Chaotic systems are often employed to produce key streams for scrambling or changing pixel values. Such key-streams can be generated using the initial values that are given to the chaotic systems. Images are created by concatenating these key streams with plaintext images. Additionally, a minor change in the seed values may result in a significantly different key stream. This property corresponds to the sensitivity to the initial condition. Chaotic systems also provide long-term random encryption by exploiting features such as state ergodicity and structural complexity.

With the use of a logistic map [10], a British mathematician named Matthews made the first formal application of chaos theory to cryptography in 1989. It has been shown in this study that chaos-based encryption is feasible. Because of the security requirements for image data, chaos is attracting the attention of researchers in image encryption [9]. The confusion–diffusion structure is used as a generic framework in the development of image encryption schemes in the majority of existing image encryption techniques. According to Shannon [11], an encryption scheme that consists of both a confusion and diffusion property can be considered a secured cryptosystem. Fridrich proposed a confusion–diffusion structure in [12] that encrypted images using the permuting pixel location and diffusing pixel value with chaos for the first time. However, as compared to permuting pixels, the permutation of bits of pixels gives more security, while the calculation cost may be larger.

### 1.2. Data Classification Using Deep Learning

Apart from securing medical images such as X-rays, it is also important to use a technique to diagnose diseases such as pneumonia with high accuracy. Several machine learning techniques have been proposed in recent years that offer an accuracy of 85–90%, which is not acceptable as it can be a source of a great loss for patients in terms of health due to misclassification between normal and pneumonia patients.

Pneumonia is an acute lung infection that causes fever, coughing, muscular pains, chills, and trouble breathing in infected individuals. The pneumonic disease has been documented throughout human history, with early Greek civilization mentioning the condition [13]. Despite our long history with the illness, pneumonia remains a significant medical problem for the global population, with millions of incidents of pneumonia-related hospitalizations and deaths globally. The World Health Organization (WHO) has stated that, in 2016, more than 700,000 children aged less than 2 years died due to pneumonia [14,15]. The death toll from pneumonia exceeded the combined tolls from malaria, AIDS, and measles in the same year. It is worth mentioning here that more than 95% of children living in backward areas with limited medical resources died of pneumonia. Therefore, a precise, low cost, and quick pneumonia diagnosis is required in such areas.

Chest X-rays are one of the most frequently utilized images for the diagnosis of diseases such as pneumonia in the liver and lungs non-invasively [16]. However, pneumonia diagnosis in chest X-rays also depends on the radiologist’s diagnostic expertise [17]. Furthermore, the trustworthiness of diagnostic results continues to face enormous obstacles. This problem arises frequently in underdeveloped countries, where the prevalence and mortality of pediatric pneumonia are much greater than the global norm, such as in the South Asian subcontinent [18]. On the other hand, it is mainly the responsibility of highly qualified and experienced doctors to identify chest X-ray images. Currently, only a few systems for medical image analysis are capable of autonomously identifying human organs or tissues [19]. The automatic classification and detection of organs based on X-ray images has enormous application potential in academic medicine. It may significantly increase the number of cases analyzed by students. Additionally, it can support the advancement of telemedicine education and help to save valuable human resources [20].

As a fundamental part of artificial intelligence, deep learning takes raw data as input and extracts the necessary features [21]. Finally, the learned features are associated with the task, automating the whole process. As a consequence, advancements in deep learning are inextricably linked to the study of the brain’s cognitive processes. Today, the most commonly used network model for deep learning is the neural network architecture. Since the idea of artificial neural networks (ANNs) was proposed in 1943, researchers have started to employ mathematical models to theoretically imitate neurons in ANNs, therefore broadening the scope of the study of artificial intelligence [22,23,24]. Deep learning algorithms, such as neural networks, have shown astounding performance in non-invasively classifying patient data problems for disease diagnoses [25,26,27,28].

## 2. Related Work

Encrypting medical images is an effective method of protecting them from attacks [29,30,31]. Several traditional encryption techniques, such as the Data Encryption Standard (DES) [32], the Advanced Encryption Standard (AES) [33], and the International Data Encryption Algorithm (IDEA) [34], were designed to protect textual data. The approaches, however, have been discovered to be incompatible with digital images due to their inherent characteristics such as high correlation and pixel redundancy and correlation [35,36]. Scholars have suggested selective encryption techniques to reduce correlation and redundancy [37,38]. For instance, some approaches encrypt critical compression coefficients [39] and others encrypt the interleaved patient information included in images [40], while others encrypt only the important bits of particular coefficients using a stream cipher [41,42,43,44]. However, such security techniques have several vulnerabilities, such as high computational time and weak security, which may also result in data loss and some false negative diagnoses [45,46,47]. Recently, Bouslimi et al. [48] proposed a combined watermarking/encryption system in Cipher-block chaining mode (CBC) to secure the integrity of medical images and create a medical image encryption technique by combining a stream cipher algorithm and two substitutive watermarking approaches [48]. They are capable of preserving an image’s integrity and authenticity. Zhang et al. [49] created a one-of-a-kind image encryption technique that combines bit-level rotation matrix permutation and block diffusion. Zhou et al. [50] developed a novel security approach that uses a hyper-chaotic system and 2D compressive sensing to concurrently encrypt and compress medical images. Zhang et al. [51] suggested a compressive sensing and pixel permutation technique for medical image encryption and compression. Additionally, it is capable of encrypting and compressing medical images concurrently. However, such approaches are not ideal for large datasets, such as X-ray image datasets, and are vulnerable to security attacks.

Numerous methodologies have been proposed to detect pneumonia using machine learning, deep learning, and transfer learning. Liang et al. [52] presented a model for the future forecasting of pneumonia non-invasively using a deep residual network (DRN) in which X-ray images are incorporated. To enhance the model training, a large dataset is used in the same field. The accuracy, recall, F1-score, and RoC values for the DRN model are 90.5%, 96.7%, 92.7%, and 95.3%, respectively. However, the DRN model is not suitable because the model accuracy is not sufficient, increasing the False Positive Rate (FPR). Morillo et al. [53] also proposed a pneumonia detection technique using machine learning in which Fourier transform analysis is used, which also helps to extract features.

Moreover, band-pass (BP) filtering is incorporated for the removal of noise from tracheal sounds. The model accuracy is 85%, which is less than the technique proposed in [54,55,56]. An automated system has been developed for the rapid diagnosis of pneumonia based on a deep sequential convolutional neural network [57]. A publicly available dataset is used in which X-ray images of healthy and pneumonia patients are included. The overall accuracy of the model is 94%, which is not acceptable to avoid FPR. In [58], Masot et al. proposed a deep learning-based technique for the prediction of COVID-1 and pneumonia. In the dataset, three different labels are used: healthy subject, subject COVID-1, and pneumonia. Based on the feature values and classification model, the desired output can be predicted. The accuracies obtained with histogram equalization and without histogram equalization are 86.1% and 88%, respectively. Ni et al. [59] also presented a deep learning approach to classify healthy, COVID-1, and pneumonia patients in which CT images are used. One of the evaluation parameters, known as accuracy, is used to gauge the performance of the model, which predicts correct results with an accuracy of 84%, which is less than the accuracy of the model proposed in [58]. Zhang et al. [60] proposed a machine learning model to detect pneumonia using chest radiography, in which a 90% rate of accuracy of true prediction is achieved, which is better than the model in [58,59]. Antin et al. [61] proposed a supervised learning approach for the diagnosis of pneumonia using machine learning algorithms in which X-ray images are incorporated into the dataset. For the classification of healthy pneumonia patients, logistic regression is used, and a 60% rate of accuracy of true predictions is achieved, which is significantly lower than other approaches and may not be suitable for the diagnosis of pneumonia. In [61], there is a lack of dimensionality reduction in the dataset, which results in less accuracy, which is 72. Jain et al. [62] presented a classification technique for the diagnosis of pneumonia in which convolutional neural networks and transfer learning are integrated.

Moreover, a dataset containing X-ray images is used and six different models are proposed for model training and testing. Such models have varying layers and achieve 92.31% accuracy, as demonstrated by model 1, which has two convolutional layers. A great deal of work has been presented in the literature in which an accuracy between 90% to 95% is achieved. A new methodology is proposed to improve the model’s accuracy, in which CNN is incorporated to classify pneumonia and healthy patients.

Apart from proposing pneumonia diagnosis using a neural network, it is also important to secure medical data so that they can be prevented form misuse. Therefore, in this research, we present a block encryption technique that combines neural networks, chaotic systems, and complementary DNA principles for securing medical data in the form of images. A novel heterogeneous chaotic system is implemented using neural networks in which random sequences are generated. Such random sequences are then utilized as inputs for the proposed encryption procedures, which include pixel position scrambling and substitution.

### Contributions of the Work

Given the current circumstances in the early WHO report, pneumonia detection and treatment are a highly demanded area of research. Our findings and contributions in this work, in particular for the identification of pneumonia, are as follows:

1. To secure the medical images (X-ray images), a neural network-based image encryption is proposed, and a comparison is made to show the effectiveness of the proposed encryption;
2. Collection of primary data from thee different hospitals in Rawalpindi/Islamabad, Pakistan;
3. Statistical and physiological features are integrated for the non-invasive diagnosis of pneumonia;
4. We developed a scheme that can reliably predict the presence of pneumonia, and it can be implemented using a smartphone application. CNN is used as a classification algorithm for the proposed approach;
5. A K-fold analysis is also used in this research to select a particular subset of the dataset, and as a consequence, the suggested model has maximum accuracy;
6. We built multiple learning models called “K-learning models” after performing the K-fold analysis. The purpose of these models is to deploy ensemble-based learning approaches.
7. For the validation of the proposed model, various metrics such as precision, recall, F1 score, and support are used. Misclassifications in the context of pneumonia detection might be exceedingly expensive in terms of human lives if a model incorrectly identifies false positives; hence, we used accuracy as mentioned in addition to the earlier measures to evaluate the proposed model. Moreover, for more validation, tuning is also performed.

The rest of the proposed work is organized as follows: Section 3 provides a description of the chaotic maps which are used in the proposed encryption scheme for data security. In Section 4, the methodology to generate the pseudo-random number is given, while Section 5 and Section 6 are devoted to the proposed encryption scheme and its evaluation, respectively. Section 7 provides the mathematics behind the proposed model with reference to the CNN. In Section 8, materials and methods regarding the dataset used in the proposed work and processing are explained. In Section 9, the proposed model is explained in detail, while in Section 10, experimental results and an analysis of the proposed work and its comparison with existing methods are given. Finally, Section 11 concludes the proposed research work.

## 3. Chaotic Maps Used in the Proposed Work

Among the several chaotic maps available in the literature, three chaotic maps—piece-wise linear chaotic map (PWLCM), logistic map, and logistic tent system (LTS)—are used in the proposed encryption scheme. In particular, such maps are incorporated to create a pseudo-random number generator.

### 3.1. Logistic Map

A one-dimensional logistic map is defined in Equation (1) [63]:

(1) $$T_{n+1}=GT_{n}\left(1-T_{n}\right)$$

where *G* is a control parameter with a value varying from 3.58 and 4. $T_{n}$ and $T_{n+1}$ are the chaotic map’s outputs/states when they iterate at *n* and n+1, respectively, and their values will lie between 0 and 1.

### 3.2. Piece-Wise Linear Chaotic Map (PWLCM)

Several linear segments that exhibit superior properties to the logistic map are used to construct the PWLCM, which is defined as follows [64]:

(2) $$T_{n+1}=F\left[T_{n}\right]=\left\{\begin{array}{ll} \left(\frac{T_{n}}{G}\right) & if\;0\leq T_{n}<G\\ \left(\frac{T_{n}-G}{0.5-G}\right) & if\;G\leq T_{n}<0.5\\ F\left[1-T_{n}\right] & if\;0.5\leq T_{n}<1 \end{array}\right\}$$

where *G* is the range-control parameter (0, 0.5). *n* and $T_{n+1}$ are the chaotic map’s outputs/states at *n* and n+1 iterations, respectively, and their values lie between 0 and 1.

### 3.3. Logistic Tent Map (LTS)

LTS is a combination of logistic and piece-wise liner maps as discribed in Equation (3).

(3) $$T_{n+1}=\left\{\begin{array}{ll} GT_{n}\left(1-T_{n}\right)+\frac{\left(4-G\right)T_{n}}{2}mod1 & if\;T_{n}<0.5\\ GT_{n}\left(1-T_{n}\right)+\frac{\left(4-G\right)\left(1-T_{n}\right)}{2}mod1 & if\;T_{n}<0.5 \end{array}\right\}$$

where *G* is the chaotic behavior control parameter in the range [0, 4], $T_{n}$ and $T_{n+1}$ are the chaotic map’s outputs/states when it iterates at *n* and n+1, respectively, and their values will lie between 0 and 1.

## 4. Proposed Pseudo-Random Number Generator

**Figure 1:**
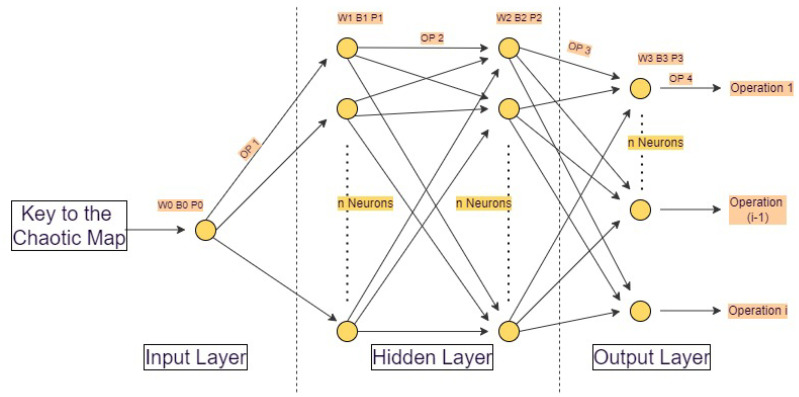
Neural network-based generator architecture.

This section describes the proposed chaotic pseudo-random generator based on neural networks. The proposed generator design is heterogeneous, implying the utilization of many chaotic maps. Figure 1 shows the neural network-based proposed generator.

Four layers comprise the proposed neural network generator: two hidden layers and two input/output layers (one input and one output). In the neural network, each neuron in the neural network generator has a transfer function defined by a chaotic map equation. The proposed architecture employs a single neuron as the input layer, which receives an encryption key with a size of 64 bits, divided into two equal sections with a size of 32 bits. The first section is devoted to the input for the neural network’s initialization cycle, while the second section serves as the output for the neural network’s initialization cycle. The two hidden layers are composed of $n_{hl}$ number of neurons, whereas the output layer is composed of $m_{ol}$ neurons.

To implement a heterogeneous chaotic system in neural network layers, multiple chaotic maps are incorporated as transfer functions. For instance, if the logistic map is empty in the first layer, the PWLCM will be used in the second layer, and so on. The weight matrices of the four layers are denoted as $W_{0}$, $W_{1}$, W2, and $W_{3}$, numbered from 0 to 3, respectively, as shown in Figure 1, and with the dimensions of 1 × 1, $n_{hl}\times 1$, $n_{hl}\times n_{hl}$, and $m_{ol}\times n_{hl}$. The layers’ bias matrices are $B_{0}$, $B_{1}$, $B_{2}$, and $B_{3}$. $P_{0}$, $P_{1}$, P−2, and $P_{3}$ are the control parameter matrices for the chaotic map transfer function. Both $B_{0}$–$B_{3}$ and $P_{0}$–$P_{3}$ are 1 × 1, $n_{hl}\times$ 1, $n_{hl}$ × 1, and $m_{ol}$ × 1, respectively. The neural network generator’s $m_{ol}$ outputs can be utilized to perform the encryption algorithm’s various operations.

A 1D Cubic map is employed to build the neural network generator. The seed values for the cubic maps are defined by the second 32 bit section of the 64 bit key.The cubic map is given in Equation (4):

(4) $$T_{n+1}=\lambda T_{n}\left(1-T_{n}^{2}\right)$$

where $T_{n}$ is the seed value for the first cycle of the chaotic function or the prior state for subsequent cycles, and $T_{n+1}$ is the chaotic function’s current state. Both $T_{n}$ and $T_{n+1}$ lie in (0, 1). λ is the chaotic control parameter, with a value of 2.59. To reveal its chaotic properties, 50 iterations are made for this map. Following that, the map’s outputs are used to initialize the initial parameters of the neural network.

In the proposed work, four outputs are utilized—i.e., $m_{ol}=4$—to generate chaotic sequences corresponding to each output:

- Pixel scrambling ($OP_{4w}$);
- Pixel substitution (PS) ($OP_{4x}$);
- Pixel bit scrambling (PNS) ($OP_{4y}$);
- ($OP_{4z}$) The total number of iterations of the chaotic neuron transfer function is updated using ($OP_{4z}$).

**Figure 2:**
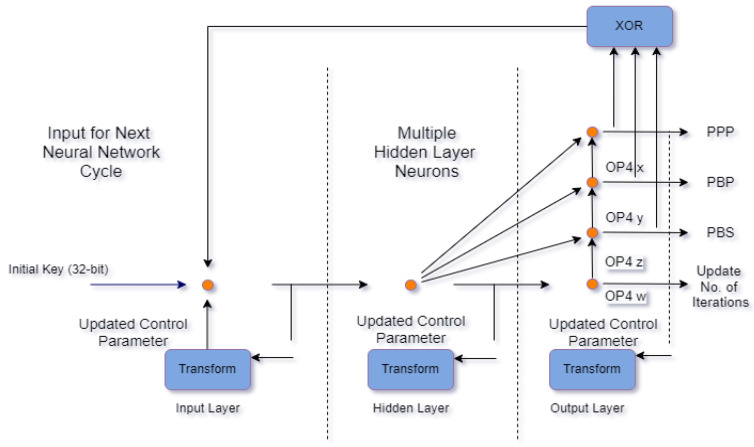
Neural network-based generator architecture.

The output of the aforementioned parameters and the working of the proposed generator are shown in Figure 2.

## 5. Proposed Cryptosystem for Medical Images

This section describes the details of the proposed cryptosystem and a novel pixel bit permutation mechanism based on DNA principles.

**Figure 3:**
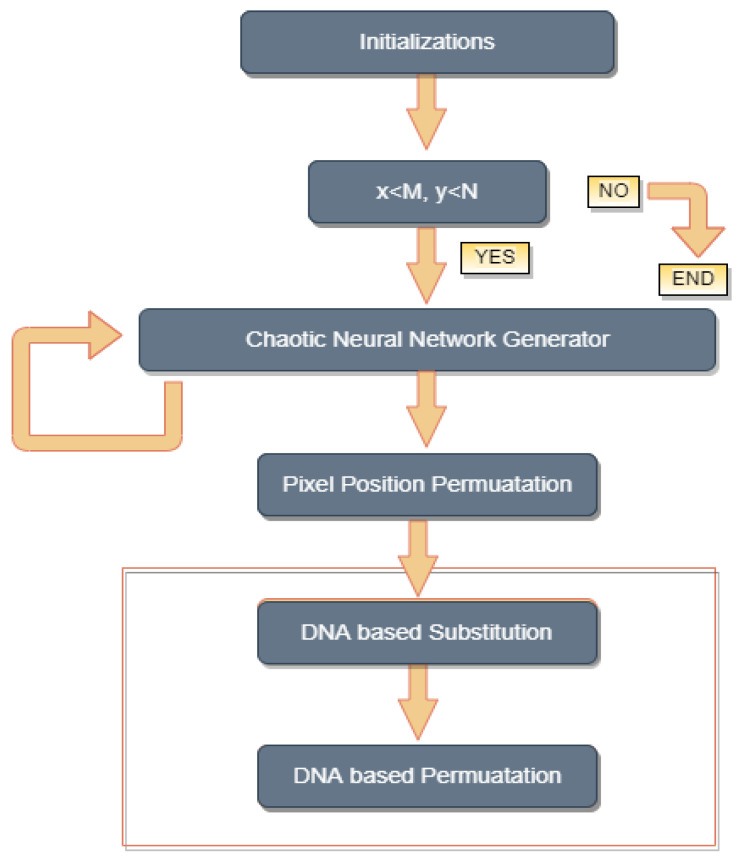
Neural network-based generator architecture.

The proposed encryption algorithm consists of two different blocks. In the first block, pixel permutation (P-box) is performed, while the second block comprises substitution and permutation, or SP-box, as shown in Figure 3.

### 5.1. P-Box

The P-box refers to the permutation-box, which is used to shuffle the locations of the image pixels by determining the pixel position.

**Figure 4:**
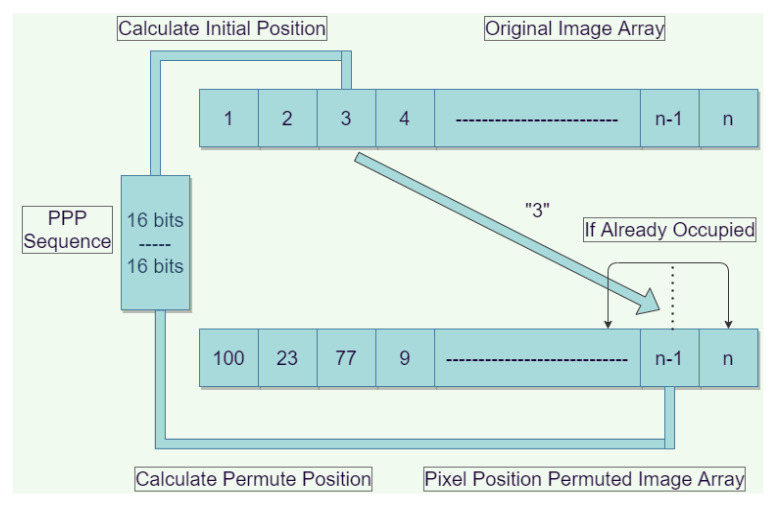
Permutation process for the proposed encryption scheme.

For an N×N image, the original positions of the image pixels are permuted using the first N-bits of the P-sequence. The last N bits are utilized to compute the chosen pixel’s target location. The number of permutation iterations is equal to the total number of pixels present in the original image (N×M), where *N* and *M* represent the number of rows and columns of the image pixels, respectively. The proposed permutation operation is shown in Figure 4, in which each pixel value is permuted according to the values preset in the P-sequence.

### 5.2. SP-Box

**Figure 5:**
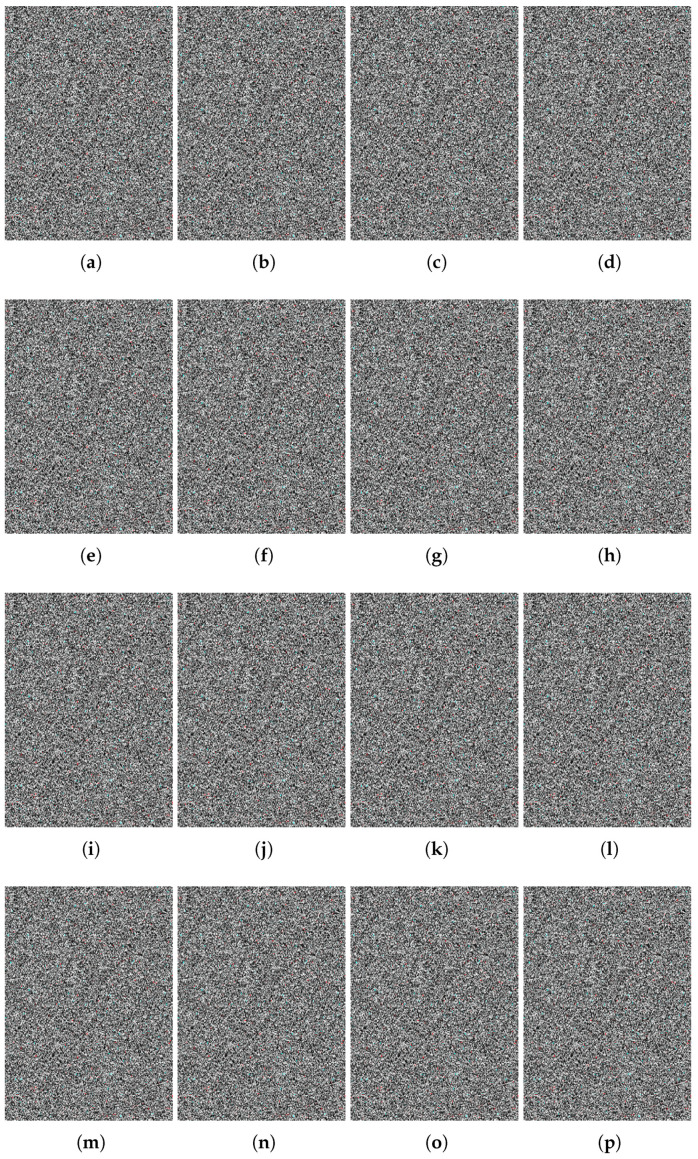
(**a**–**p**) Encrypted images corresponding to normal and pneumonia patients’ X-ray images.

**Figure 6:**
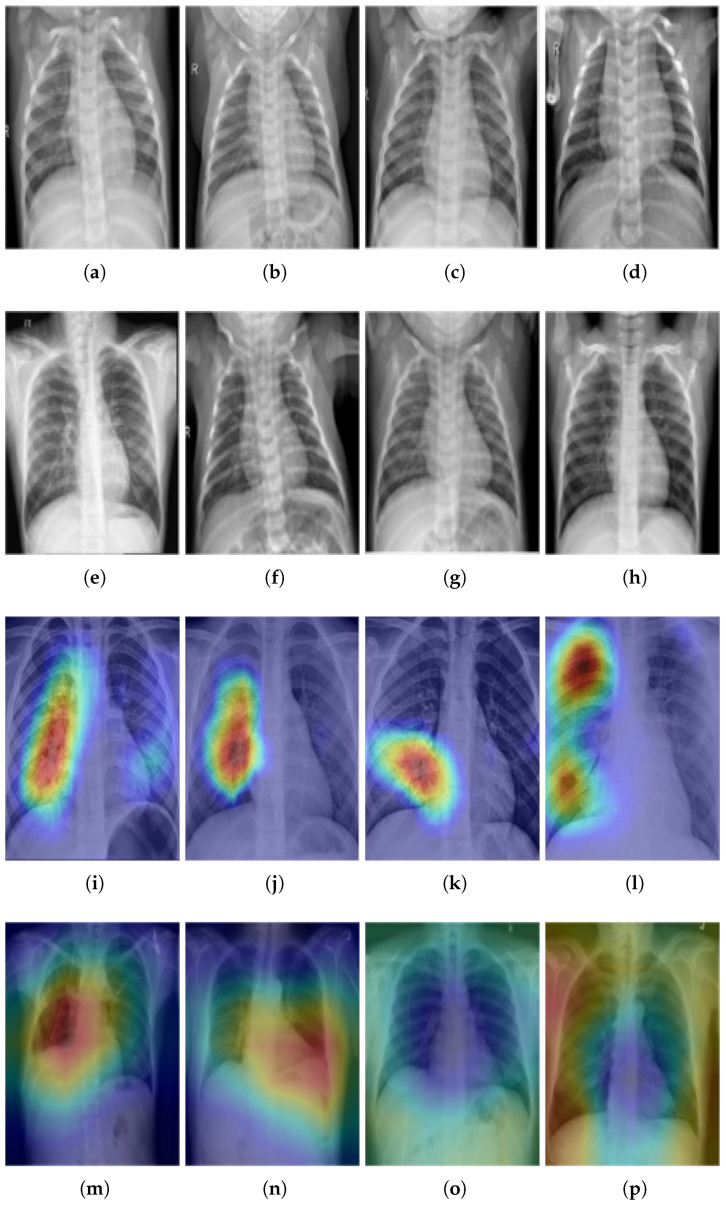
(**a**–**h**) X-ray images of normal patients, (**i**–**p**) X-ray images of pneumonia patients.

In this step, the input is the scrambled pixels, which are permuted in the P-box. The SP-box contains two operation: one is substitution and the other is permutation. Such operations are performed on each pixel one by one; i.e., the pixel value is substituted with the new value followed by the permutation of the substituted pixel value. The permutation operation is then applied on bits of the bi-plane, which are extracted from the plane text image. The operations in the SP-box are repeated until the substitution and permutations of the last pixel value to obtain the final encrypted image. Such encrypted images are shown in Figure 5 and their corresponding plaintext images are shown in Figure 6.

## 6. Proposed Encryption Scheme Evaluation

To gauge the performance of the proposed encryption algorithm, the following parameters are under consideration:

- Mean square error (MSE);
- Peak signal to noise ratio (PSNR);
- Entropy;
- Correlation;
- Energy;
- Contrast;
- Execution time.

The detail of such security parameters can be seen in [65,66,67]. Table 1 shows the statistical value of the security parameters calculated for the evaluation of the proposed cryptosystem for medical images.

As with the MSE, the contrast of the encrypted image should be high as much as possible, while the correlation, energy, and execution time should be low for strong encryption. However, the entropy value must be close to 8.00 for an 8 bit encrypted image. It can be seen from Table 1 that the values for the security parameters for the proposed work are much better than existing schemes.

### Noise Attack Analysis

**Figure 7:**
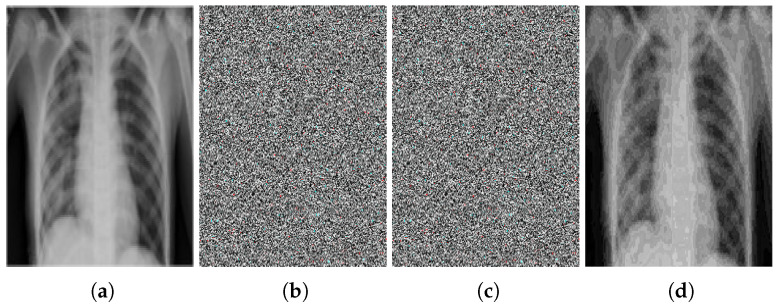
Noise attack analysis: (**a**) plaintext image, (**b**) encrypted image, (**c**) noisy encrypted image, (**d**) decrypted image.

To check the robustness of the proposed encryption algorithm against noise attack, salt and pepper noise is added to the encrypted image and decrypted with the decryption algorithm. It is found that the information in the decrypted image, which is reconstructed from the noise-encrypted image, is clearly seen, as can be seen in Figure 7. There is some noise in the decrypted image, but the content of the plaintext image is visible.

## 7. Convolutional Neural Networks (CNN)

**Figure 8:**
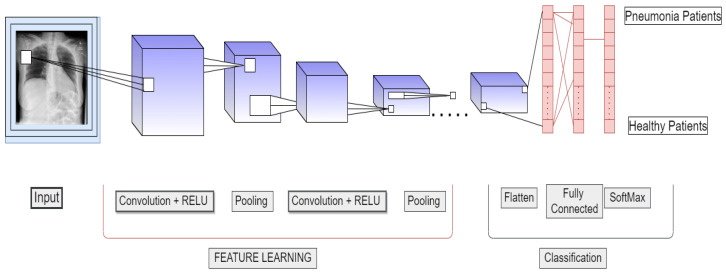
CNN architecture.

In recent years, CNN has dominated the machine vision domain. A CNN consists of several hidden, input, and output layers. Convolutional, pooling, fully connected, and normalizing layers are common hidden layers in CNNs. For increasingly sophisticated models, more layers might be employed. Examples of a standard CNN are illustrated in Figure 8.

The vector calculus and chain rule may be used in the CNN learning process [74]. Let *T* be any scalar (i.e., *T* ∈ *R*) and *x* ∈ R${}^{W}$ be a vector. The partial derivative of *T* with regard to *x* will be

(5) $$\frac{\partial T}{\partial x}=\frac{\partial T}{\partial x_{j}}$$

The size of $\frac{\partial T}{\partial x}$ is same as *x*, and the *i*^th^ element is $\frac{\partial T}{\partial x}$. Also, $\frac{\partial T}{\partial x^{Q}}$ = ($\frac{\partial T}{\partial x}{)}^{Q}$. Moreover, it is presumed that *N* ∈ $R^{M}$. In this case, the partial derivative of *T* with regard to *x* will be

(6) $${\left(\frac{\partial x}{\partial N^{Q}}\right)}_{jk}=\frac{\partial x_{j}}{\partial N_{j}}$$

The derivative is represented by Equation (6), which is a P×S matrix with the entry at the intersection of the *j*^th^ row and *k*^th^ column. From this, the chain rule can be incorporated to calculate the following:

(7) $$\frac{\partial T}{\partial N^{Q}}=\left(\frac{\partial T}{\partial N^{Q}}\right)\left(\frac{\partial x}{\partial N^{Q}}\right)$$

A cost or loss function may be used to calculate the difference between a CNN’s $N^{L}$ forecast and the outcome *t*, $N^{1}$→$M^{1}$, $N^{2}$→…, $N^{L}$→$S^{L}$ = *T*, using a simplistic loss function *T* = |t−$N^{L}$${|}^{2}$. However, more complicated functions are often used. A prediction’s output can be thought of as argmax ${}_{i}$. $xL_{i}$. Convolution may be stated as follows:

(8) $$x_{j^{l+1}.k^{l+1},d}=\sum_{j=0}^{P}\sum_{k=0}^{M}\sum_{d=0}^{D}g_{j.k.d}\times N_{j^{l+1}+j,k^{l+1}+k,d}^{L}$$

The size of the filter *g* is ($P\times M\times D^{l}$); thus, the spatial size of the convolution will be ($P^{l}-P+1)$ × ($M^{l}-M+1$) with D slides, which means *y*($x^{l+1})$ in $R^{P^{l+1}\times M^{l+1}\times D^{l+1}}$, $P^{l+1}$ = $P^{l}-P$ + 1, $M^{l+1}$ = $M^{l}-M+1$, $D^{l+1}$ = *D*.

The probability of each symbol—for example, *S* ∈ {1, 2, 3, … *S*} for each training sample—is calculated using Equation (9).

(9) $$P\left(S|N\right)=\frac{exp(TS)}{\sum_{j}^{S}exp\left(T_{j}\right)}$$

where *T* is a non-normalized log probability. The cross entropy for this model can be computed as

(10) $$Ent=\sum_{s=1}^{S}log\left(p\left(S\right)q\left(S\right)\right)$$

Because cross-entropy loss is differentiable in terms of logistics, $T_{S}$, it may be used to gradient-train deep models. The range is between −1 and +1, where the simple form of the gradients is $\frac{\partial l}{\partial_{T_{S}}}$. Typically, when cross-entropy is minimized, the log-likelihood of the correct label is maximized. A CNN architecture analyzes a distribution over labels independent of training instances u(S) with a smooth parameter ζ, since this may create some overfitting problems, where for a training example, q(S|N) = $\sigma_{S,N}$ is replaced as follows [75]:

(11) $$q^{{}^{\prime }S|N}=\left(1-\zeta \right)\sigma_{S,N}+\zeta \mu \left(S\right)$$

where $q^{{}^{\prime }S|N}$ is a mixture of $q^{S|N}$ with weight 1 − ζ and the fixed distribution μ(S) with weight ζ. When label-smoothing regularization is applied with μ(S) = $\frac{1}{S}$, $q^{{}^{\prime }S|N}$ becomes

(12) $$q^{{}^{\prime }S|N}=\left(1-\zeta \right)\sigma_{S,N}+\frac{\zeta }{S}$$

Alternatively, this can be expressed in the form of entropy as follows:

(13) $$P\left(q^{\prime },v\right)=-\sum_{s=1}^{K}log\left(p\left(s\right)\right)q^{\prime \left(s\right)}=\left(1-\zeta \right)P\left(q^{\prime },v\right)+\zeta P\left(u,p\right)$$

Thus, the label-smoothing regularization is equivalent to replacing a single cross-entropy loss H(q,v) with two losses, H(q,v) and H(u,v), with the second loss penalizing the deviation of the predicted label distribution *v* from the prior *u* with a relative weight of $\frac{\zeta }{\left(1-\zeta \right)}$, which is equivalent to computing the Kullback–Leibler divergence. A further mathematical formulation of CNN architecture can be seen in [76,77].

### 7.1. Transfer Learning

Transfer learning is a type of machine learning that involves training to construct a model for prediction tasks such as COVID-19 and liver disease forecasting [78,79]. It is a circumstance in which information obtained in one context is utilized to maximize performance in another [52,80,81]. Transfer learning is often utilized when the new dataset on which the pre-trained model is trained is smaller than the original dataset. This research work presents a strategy for repurposing an Inception-v3 model trained on a base dataset (ImageNet) to learn (or transfer) features for training on a new dataset (CIFAR-10 and Caltech Faces) [54]. Transfer learning enables us to start with the features learned on the ImageNet dataset and adapt them, as well as the model’s structure, to the new dataset/task, rather than beginning the learning process from scratch with random weight initialization.

### 7.2. Fine Tuning

The proposed model is tuned to recognize 1000 generic object classes listed as part of the ImageNet hierarchy [82].

The concept of fine-tuning a network is based on the idea of transfer learning [83]. We train a CNN to learn features for a large domain by optimizing the classification function for that domain’s error minimization. After replacing the classification function, the network is adjusted again to minimize the error in a more specific domain. This configuration moves the network’s features and properties from the wide domain to the specialized domain.

The CNN classification function, known as softmax, is used to calculate the probability of each of the 1000 classes in the ImageNet dataset. To commence the fine-tuning procedure, existing softmax classifier values are deleted and replaced with new random values, and an updated softmax classifier is then trained from scratch by incorporating the back-propagation algorithm in which a large amount of pneumonia patient data is used.

**Figure 9:**
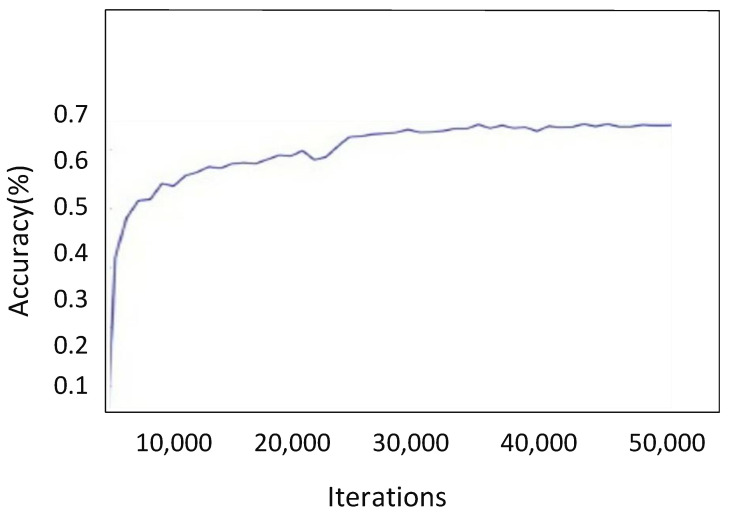
Illustration of increase in accuracy by increasing the number of iterations.

It is crucial to precisely calibrate the learning rates of each layer before initiating the back-propagation process for fine-tuning. In this research, we boosted the learning rate of the top classification layer to 10 and dropped it to 0.1 for the next seven layers. Five thousand rounds of the back-propagation approach were employed, using stochastic gradient descent to optimize the network parameters (SGD). The accuracy of image classification in a validation set develops throughout the fine-tuning process, as seen in Figure 9. Accuracy significantly increased throughout the first rounds and stabilized at roughly 40,000 iterations.

## 8. Materials and Methods

The proposed non-invasive model for pneumonia diagnosis complements the conventional diagnostics tools/mechanisms and enables medical practitioners to diagnose pneumonia with better reliability and accuracy. There are 5300 verified chest X-ray images in the dataset used in the proposed research. The X-ray images for the healthy and pneumonia patients and the distribution of the data are shown in Figure 6.

The images are divided into two groups: a training set and a testing set of patients. All chest X-ray imaging was performed as part of the patients’ regular medical treatment. To analyze chest X-ray images, all chest radiographs were first reviewed for quality control, with any scans that were poor quality or illegible being removed. After that, the diagnoses for the images were rated by two experts before being approved for use in the AI system. Finally, a third expert evaluated the assessment set to make sure there were no grading problems.

Because the images are of varying sizes and quality, attributes such as sharpness, contrast, and brightness levels are varied for practically all of them. Pneumonia can be diagnosed with the help of X-rays. According to findings on X-ray images, organic causes of pneumonia include uneven mucosal surfaces and increased luminal fluid. Different patterns may be seen in X-ray images while looking for pneumonia. As seen in Figure 6, healthy and normal patients and pneumonia-infected individuals exhibit various patterns in their X-ray images, as seen in Figure 6. From X-ray images, many patterns may be used to identify pneumonia symptoms. Further information on these symptoms based on X-ray pictures is given in [84].

There are various parameters and symptoms to examine when attempting to diagnose a person with pneumonia, including cough, fever, chest pain, flu, low energy, sweating and shaking, chills, shortness of breath, fatigue, loss of appetite, and headache. However, not all symptoms appear in every pneumonia patient, and the severity of each symptom may vary from patient to patient.

### Data Representation

**Figure 10:**
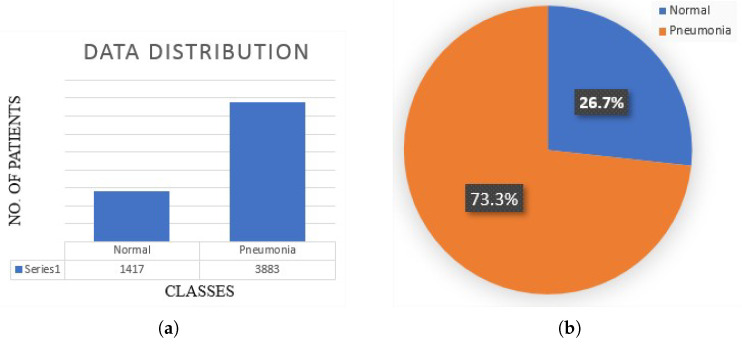
Distribution of dataset: (**a**) Population of normal and pneumonia patrinets in bar graph (**b**) Percentage of normal and pneumonia patients in pie chart.

The data used in the proposed work are in the form of X-ray images, which may be used to identify infections in the liver and lungs, as well as pneumonia [85]. The data percentages of pneumonia and healthy patients are shown in Figure 10.

**Figure 11:**
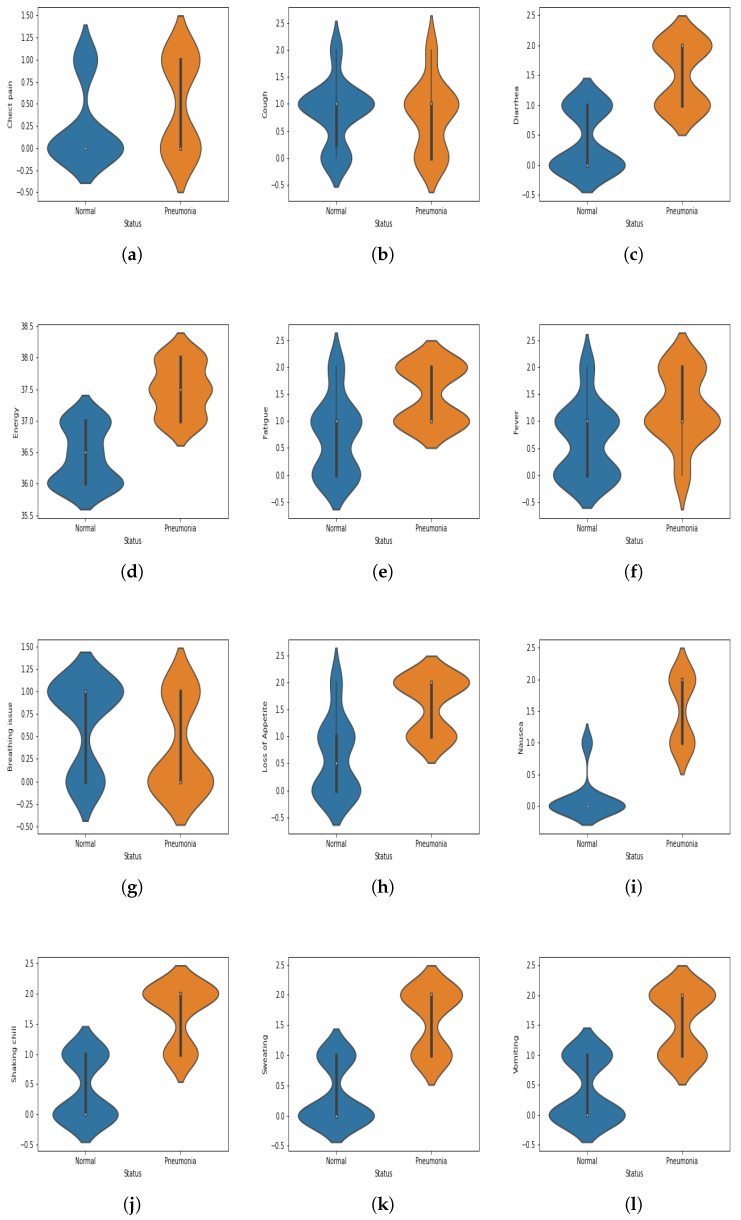
(**a**–**l**) Violin plots corresponding to each feature used in the dataset.

Several physiological symptoms are considered for pneumonia diagnosis. Such symptoms are assigned a specific numerical value, as shown in Table 2. The numerical data are defined as follows: if a person is suffering from fever, the statistical value for the scenario will be 1, and vice versa. The values 1 (yes) and 0 (mo) are the indication of the presence and absence of symptoms, respectively. All the numeric values for each symptom are displayed in Table 2. Table 3 shows the data for only 60 patients. Moreover, for the better visualization of the dataset, violin plots are given in Figure 11. Apart from the physiological features, several statistical features are also used to build the proposed model. The details of the statistical features can be seen in [86].

## 9. Model Selection, Training and Evaluation

**Figure 12:**
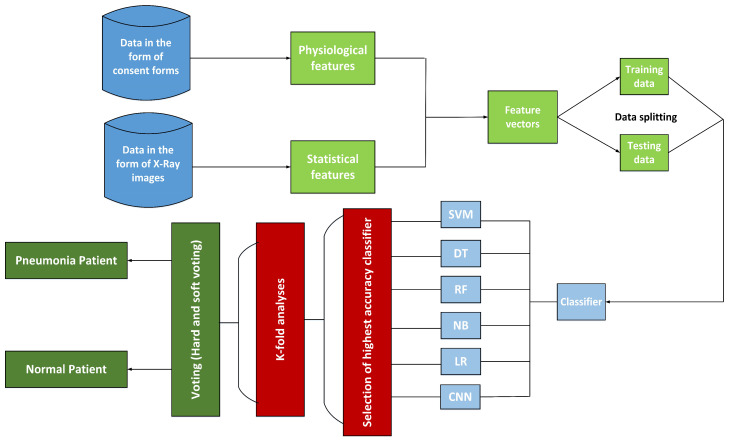
Flow diagram of the proposed work.

The goal of the proposed work is to classify healthy subjects and pneumonia disease subjects, differentiating them from each other. To achieve this classification, the classification performance is analyzed. The block diagram of the proposed work is given in Figure 12. The step by step explanation of the proposed model is presented below.

1. The collection of data was in the form of (X-ray) images and consent forms. Different X-ray images varied with size; for example, A×B, where *A* and *B* denote the rows and columns of pixels, respectively.
2. Statistical features were extracted from the X-ray images using CNN, which extracted features through the filters available in different layers. Initial layer filters were responsible for extracting low-level features, while higher layers filters were responsible for extracting high-level features. Such extracted features were then forwarded to the classifier for the decision.
3. Split features were extracted from X-ray images by observing patterns. For example, the patterns shown on the X-ray images of healthy individuals varied from the patterns that appeared on the X-ray images of pneumonia-infected patients. Here, CNN extracted different features/patterns and took a decision based on the extracted features/patterns. Pneumonia images are different from those of normal patients. From Figure 6i–p, one can see different colors, showing a sign of abnormality in the patient. However, the X-ray of the normal patients does not show color variation (see Figure 6a–h).
4. Distinct feature vectors (*X.Vs*) for each X-ray image were made: X.V = $X_{1}$, $X_{2}$, $X_{3}$ … $X1_{4}$.
5. The given matrix (X.Vs) represents only those feature vectors created from statistical features extracted from the X-ray images. Such features can be expressed in a single data set, as shown in Equation (14).
  (14) $$\mathrm{Feature}\;\mathrm{vectors}\;\left(X.\mathrm{Vs}\right)=\left(\begin{array}{c} X.V_{1}=X_{1},X_{2},X_{3}\ldots X_{14}\\ X.V_{2}=X_{1},X_{2},X_{3}\ldots X_{14}\\ X.V_{3}=X_{1},X_{2},X_{3}\ldots X_{14}\\ \vdots \\ X.V_{n}=X_{1},X_{2},X_{3}\ldots X_{14} \end{array}\right)$$

The dataset was split into two sections: one for training and the other for testing. The training data were drawn from a collection of healthy and pneumonia-infected subjects, and the model was trained using CNN. In total, 80% of the dataset was chosen at random for training purposes, with both healthy and contaminated data types taken into account, as given in Equation (15). The precise proportion of data allotted for training and testing might vary. Previously, CBC (complete blood count) determined the type and amount of white blood cells in the body; findings may indicate the presence of an illness. Blood tests for potassium, sodium, and other chemistries (BMP) have been used to evaluate the severity of a disease. This is a non-invasive method to examine the human body, and safety measures must also be adhered to while performing the whole procedure; otherwise, it will be harmful to human beings. Machine learning approaches are used in the proposed study to overcome the weaknesses in CBC and BMP for the real-time identification or diagnosis of pneumonia.

(15) $$\left\{\begin{array}{cc} \mathrm{if}\;\mathrm{Testing}\;\mathrm{data}\;\mathrm{samples} & \;T\;=\;100\\ \mathrm{Training}\;\mathrm{data}\;\mathrm{sample} & \;(\mathrm{Total}\;F.\mathrm{Vs})\;-\;\left(100\right) \end{array}\right.$$

CNN was applied to the proposed mode to classify pneumonia and healthy subjects. Moreover, several ML algorithms such as decision tree, naive Bayes and random forest were also tested on the proposed work. The purpose of using other classifiers/Ml algorithms was to compare the results of the ML algorithm with the deep learning algorithm. In this work, the performance of CNN is better than other ML algorithms, as can be seen in Table 4. Therefore, CNN is selected in this research work due to its higher performance.

Moreover, to improve the accuracy of the proposed model, we applied transfer learning and fine tuning. After fine tuning, the model accuracy was found to be 96%. Further, to enhance the proposed model, K-fold analysis and voting techniques were also incorporated, and an accuracy of 97.7% was successfully achieved. The detailed results and analysis are given in Table 4 and Table 5.

### 9.1. K-Fold Analysis

The statistical approach of cross-validation is used to estimate the skill of machine learning models. We initially shuffled our dataset in k-fold cross-validation such that the order of the inputs and outputs was perfectly random. These steps were performed to ensure that our information was not skewed in any manner. The dataset was then divided into k equal-sized sections. We employed 10-fold cross-validation in this analysis.

As a result, the very first step was to shuffle and divide our data into 10 folds. Then, in each k interaction, we utilized one fold for testing and calculating the empirical square loss and the remaining nine to train our model. As a result, we chose a different fold for testing each time that we started a new interaction. This ensured that each of the k parts was only utilized for testing once. The values for the parameters such as accuracy, precision, recall, and F-1 score are listed in Table 5.

In the proposed work, five different values of K were selected (K = 25, K = 30, K = 35 and K = 40) to generate four different K-models in order to apply the voting techniques, which are explained in the next sections.

### 9.2. Voting Techniques

The Ensemble Voting Classifier is a meta classifier that combines comparable or excellent machine learning classifiers for classification and detection. “Hard” and “soft” voting are carried out via the Ensemble Voting Classifier.

#### 9.2.1. Hard Voting

**Figure 13:**
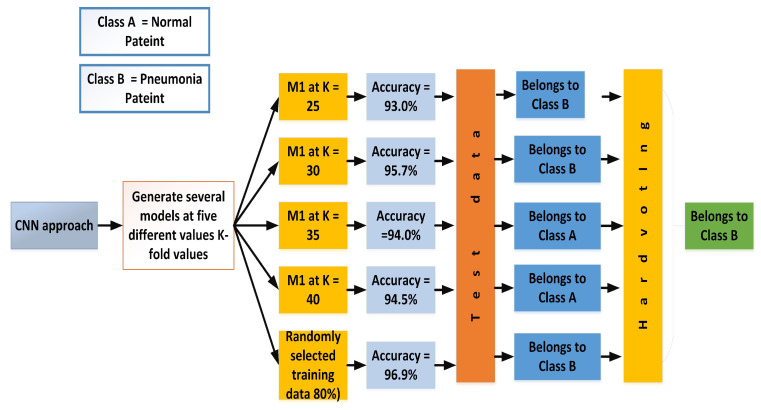
Classification after applying hard voting.

The simplest case of majority voting is hard ensemble voting. This technique works on the majority votes. For instance, in the proposed research, to increase the model’s accuracy, dif k using k-fold analysis classifies the event into classes *A* and *B* based on the majority votes. In Figure 13, the accuracies of all the k-models are displayed, and it can be seen that the majority votes in favor of class *A*. Therefore, after applying hard voting, an upcoming sample will fall in class *A*.

#### 9.2.2. Soft Voting

**Figure 14:**
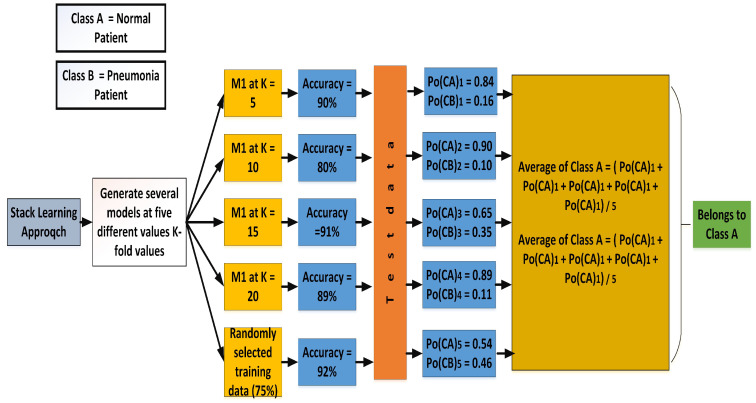
Classification after applying hard voting.

In soft voting, the classes are predicted based on the classifier’s predicted probability p. In Figure 14, different probabilities of a particular type occurring through an individual k-model are displayed. Mathematically, the probability of either class and the assignment of the class to the upcoming sample can be calculated using Equations (16) and (17).

(16) $$\mathrm{For}\;\mathrm{class}\;A=\frac{Po{\left(E_{A}\right)}_{1}+Po{\left(E_{A}\right)}_{2}+Po{\left(E_{A}\right)}_{3}+\ldots +Po{\left(E_{A}\right)}_{N}}{N}$$

(17) $$\mathrm{For}\;\mathrm{class}\;B=\frac{Po{\left(E_{A}\right)}_{1}+Po{\left(E_{A}\right)}_{2}+Po{\left(E_{A}\right)}_{3}+\ldots +Po{\left(E_{A}\right)}_{N}}{N}$$

where $Po(E_{A})$ and $Po(E_{B})$ is the probability of events *A* and *B* occurring, respectively.

According to the calculated probabilities, Equations (16) and (17) become

(18) $$\mathrm{For}\;\mathrm{class}\;A=\frac{0.84\;+\;0.90\;+\;0.65\;+\;0.89\;+\;0.54}{5}=76.4$$

(19) $$\mathrm{For}\;\mathrm{class}\;B=\frac{0.16\;+\;0.10\;+\;0.65\;+\;0.89\;+\;0.46}{5}=23.6$$

## 10. Experimental Setup

The presented model was developed in Python, and the computer system on which it was run had the following specifications: 8 GB RAM, Windows 8, and a graphics card. Furthermore, Python 3.7 uses several libraries such as pandas, Matplotib, Numpy, Seaborn, Tensorflow, Sklearn, CV2, Image Data, and transfer learning.

### 10.1. Results and Discussion

To analyze the model’s performance in diagnosing pneumonia, it was necessary to examine the performance of classifiers using quantitative measures to evaluate their effectiveness. Therefore, metrics such as precision, recall, F1 score, and support were used to assess the proposed model. Following that, the results of the TP (True Positive), TN (True Negative), FP (False Positive), and FN (False Negative) tests were used to create the five performance measures indicated in the confusion matrix.

### 10.2. Confusion Matrix

**Figure 15:**
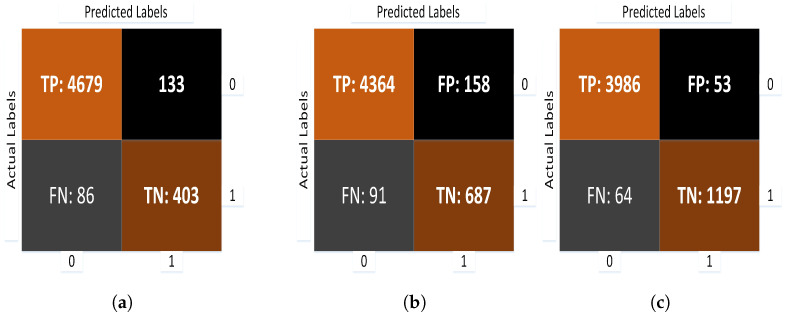
Confusion matrices for CNN, transfer learning, and the fine tuning-based proposed model. (**a**) Confusion matrix for CNN model. (**b**) Confusion matrix for transfer model. (**c**) Confusion matrix for fine tuning model.

**Figure 16:**
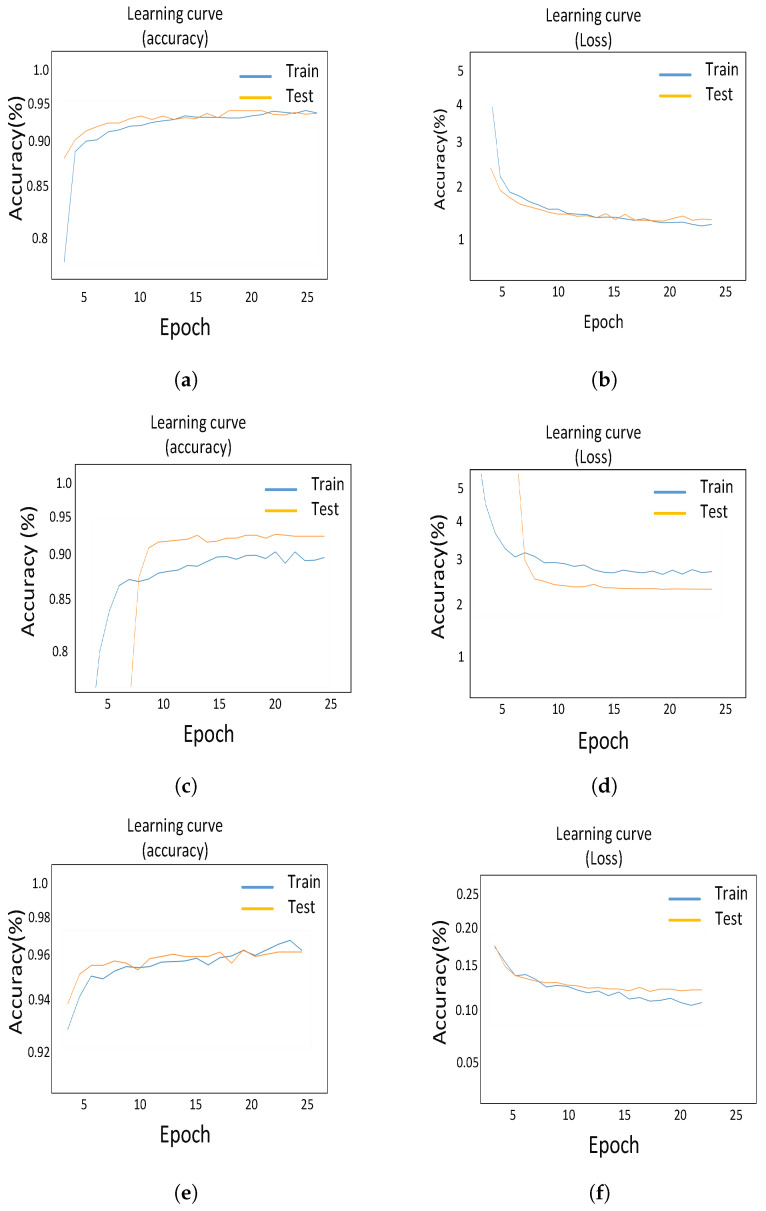
Confusion matrices for CNN, transfer learning and the fine tuning-based proposed model. (**a**) Accuracy curve for CNN model. (**b**) Loss curve for CNN model. (**c**) Accuracy curve for transfer learning model. (**d**) Loss curve for transfer learning model. (**e**) Accuracy curve for fine tuning model. (**f**) Loss curve for fine tuning model.

The classifier’s accuracy and error can be represented via the confusion matrix. This describes the model’s performance on test images when the real values are known and summarizes the output results. Figure 15 shows the confusion matrix for the proposed models in which CNN, transfer learning and fine tuning are applied:

The suggested CNN-based model (model-1) has a training and validation accuracy of 95.1% and of 95.3%, respectively, while model-1 has training and validation losses of 0.18% and 0.15%, respectively. Transfer learning improved the training and validation accuracy of model-1 to 93.6% and 95.5%, respectively. While the training and validation losses are 1.8% and 1.3% at this level, Finally, the transfer learning-based model is fine-tuned, and it achieves a training and validation accuracy of 96.5% and 97.1%, respectively. Similarly, when fine tuning is used, the training and validation losses are reduced to 0.8% and 1.2%, respectively. This impact can be seen in Figure 16.

### 10.3. Receiver Operator Characteristic (ROC) Curve

**Figure 17:**
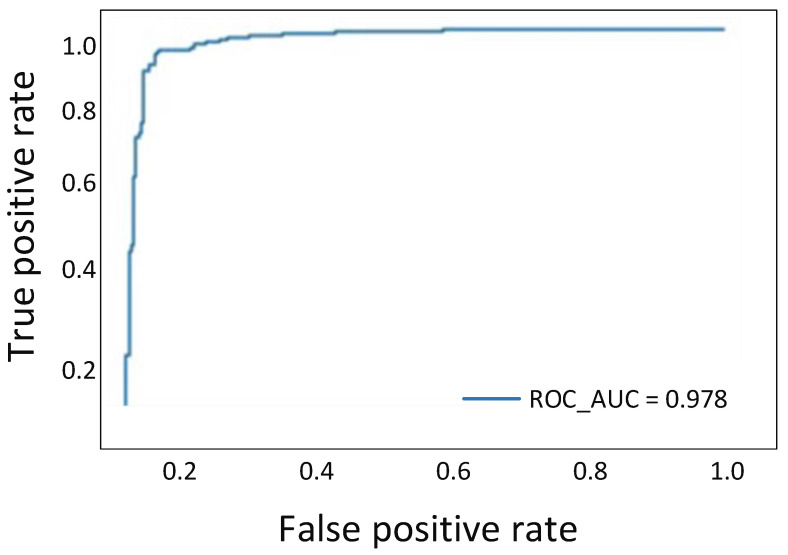
Flow diagram of the proposed work.

An ROC curve represents a binary classifier’s diagnostic ability. It originated in signal detection theory but has since been used in various fields, including medicine, radiography, natural disasters, and machine learning. The receiver operating characteristic (ROC) curve illustrates the trade-off between sensitivity (or TPR) and specificity (1–FPR). Classifiers that produce curves closer to the top-left corner perform better. From Figure 17, it can be seen that the ROC curve for the proposed work is closer to the desired value.

## 11. Conclusions

The proposed work is intended to secure X-ray images and identify pneumonia based on neural networks. To ensure that the patients’ X-ray images are not used by any unauthorized person, they are secured using chaos and neural networks. Moreover, a neural network-based model is proposed for the pneumonia diagnosis with high accuracy. In the diagnosis of this disease, false positive events are more harmful than false negative events. Hence, a deep learning system algorithm such as CNN is incorporated in the proposed work to increase the true positive rate. Moreover, to further increase the accuracy of the proposed model, transfer learning and fine tuning are also incorporated. Numerous evaluations of our suggested model, including testing through the analysis of k-fold experimentation and performance comparisons of other algorithms, helped us to select the best-suited deep learning method for completing the desired task. From the experimentation and analysis, it can be seen that the accuracy of the proposed work is 97%. Moreover, the proposed methodology works better when compared with the machine learning and deep learning-based existing models.

The proposed work is based on a deep learning algorithm. The accuracy of the proposed work is acceptable. However, it can be improved by the use of hybrid AI algorithms in the future.

## Figures and Tables

**Table 1:** Statistical security analysis of the proposed and existing encryption schemes.

				**Proposed Work**			
**Encrypted X-ray Images**	**MSE**	**PSNR**	**Entropy**	**Correlation**	**Energy**	**Contrast**	**Execution Time (s)**
Normal patient X-ray-1	258	18	7.9992	0.0001	0.154	9.2413	0.021
Normal patient X-ray-2	259	15	7.9991	−0.0054	0.0156	10.7891	0.020
Normal patient X-ray-3	260	16	7.9988	0.0010	0.0155	10.1584	0.025
Normal patient X-ray-4	251	16	7.9991	0.0001	0.0155	10.7914	0.027
Pneumonia patient X-ray-1	251	19	7.9991	−0.0035	0.0152	10.7341	0.029
Pneumonia patient X-ray-2	256	17	7.9997	0.0006	0.0151	10.7982	0.030
Pneumonia patient X-ray-3	251	16	7.9992	−0.0015	0.0155	10.1351	0.022
Pneumonia patient X-ray-4	260	15	7.9990	0.0004	0.0154	10.7546	0.025
Average	260	15	7.9990	0.0004	0.0154	10.7546	0.025
				**Comparison**			
**Existing** **Schemes**	**MSE**	**PSNR**	**Entropy**	**Correlation**	**Energy**	**Contrast**	**Execution Time (s)**
Ref. [68]	249	20	7.9953	−0.0015	0.0156	9.9882	1.361
Ref. [69]	248	25	7.9959	0.0006	0.0155	9.9783	1.399
Ref. [70]	242	20	7.9981	0.0002	0.0151	9.9985	2.798
Ref. [71]	250	20	7.9981	0.0006	0.0155	9.6570	2.331
Ref. [7]	249	23	7.9925	−0.0075	0.0160	9.9944	2.978
Ref. [72]	248	26	7.9944	−0.0050	0.0159	9.6986	2.036
Ref. [73]	247	21	7.9972	0.0009	0.0158	9.9973	2.971

**Table 2:** Representation of dataset.

Value Assigned	Cough	Fever	Breathing Issues	Chest Pain	Loss of Appetite	Energy	Fatigue	Vomiting	Nausea	Sweating	Shaking Chill	Diarrhea
Yes	1	1	1	1	1	1	1	1	1	1	1	1
No	0	0	0	0	0	0	0	0	0	0	0	0
High intense symptom	2	2	2	2	N/A	N/A	N/A	2	2	2	2	N/A

**Table 3:** Data for first 60 patients.

Value Assigned	Cough	Fever	Breathing Issues	Chest Pain	Loss of Appetite	Energy	Fatigue		Vomiting	Nausea	Sweating	Shaking Chill	Diarrhea	
P-1	0	0	1	1	1	0	0	0	0	0	0	0	0	Normal
P-2	1	0	1	0	0	1	0	0	0	0	0	0	0	Normal
P-3	0	0	0	0	1	0	0	1	0	1	0	0	0	Normal
P-4	1	0	0	1	0	0	1	1	0	1	0	1	0	Normal
P-5	1	0	1	0	0	0	0	0	0	0	0	1	0	Normal
P-6	1	0	1	0	0	1	1	1	0	0	0	0	0	Normal
P-7	1	0	1	0	0	1	1	0	0	0	0	0	0	Normal
P-8	1	0	1	0	1	0	1	1	0	1	0	0	0	Normal
P-9	1	0	1	0	1	0	0	0	0	1	0	0	0	Normal
P-10	1	1	1	0	1	1	1	1	0	0	1	1	0	Normal
P-11	1	0	1	0	1	0	1	1	0	0	1	1	0	Normal
P-12	1	0	1	0	1	0	1	1	0	0	1	0	0	Normal
P-13	1	1	1	0	1	0	0	0	0	0	1	0	0	Normal
P-14	1	1	1	0	1	0	1	1	0	1	1	0	0	Normal
P-15	1	1	0	0	0	1	1	1	0	1	1	0	0	Normal
P-16	0	1	0	0	0	0	0	0	0	0	1	1	0	Normal
P-17	0	1	0	0	0	0	0	0	1	0	1	1	0	Normal
P-18	0	1	0	0	0	0	0	1	0	0	1	1	0	Normal
P-19	0	1	0	1	1	0	0	1	0	0	1	1	0	Normal
P-20	0	1	0	1	0	1	0	1	0	0	1	1	0	Normal
P-21	1	0	1	0	0	0	0	0	0	1	1	0	1	Normal
P-22	1	0	1	0	0	0	0	0	0	1	0	0	0	Normal
P-23	1	0	1	0	0	0	0	0	0	0	0	0	0	Normal
P-24	1	1	0	1	1	0	1	1	1	0	0	1	1	Normal
P-25	1	1	0	1	1	1	1	1	0	0	0	1	1	Normal
P-26	1	1	0	1	1	0	1	1	0	0	0	1	0	Normal
P-27	1	1	1	0	1	0	2	1	0	0	0	0	0	Normal
P-28	0	0	1	0	0	0	2	0	0	0	0	0	0	Normal
P-29	0	1	1	0	0	1	2	0	0	0	0	0	0	Normal
P-30	0	1	1	0	1	0	2	0	0	0	0	0	0	Normal
P-31	0	1	1	0	0	0	2	0	0	0	0	0	0	Pneumonia
P-32	0	1	1	0	1	1	1	0	0	0	1	0	1	Pneumonia
P-33	0	0	1	0	0	1	1	0	0	0	1	0	0	Pneumonia
P-34	1	0	1	0	1	1	1	0	0	0	1	0	0	Pneumonia
P-35	1	0	1	0	0	0	1	0	0	0	1	0	1	Pneumonia
P-36	1	0	1	0	2	0	2	0	0	0	1	1	0	Pneumonia
P-37	1	1	1	0	0	1	1	1	0	0	0	1	1	Pneumonia
P-38	1	0	1	0	1	1	2	1	0	1	0	0	0	Pneumonia
P-39	1	1	1	0	0	0	1	1	0	1	0	1	1	Pneumonia
P-40	1	2	1	0	2	1	0	0	0	1	0	1	0	Pneumonia
P-41	2	2	1	1	2	1	0	0	0	1	1	1	1	Pneumonia
P-42	2	0	0	1	2	0	0	0	0	0	0	0	0	Pneumonia
P-43	2	1	0	0	0	1	0	0	1	1	1	0	1	Pneumonia
P-44	2	0	0	1	1	1	0	0	1	1	0	0	0	Pneumonia
P-45	1	2	0	0	1	1	0	0	1	0	0	0	1	Pneumonia
P-46	1	1	1	1	2	0	1	0	1	1	0	1	1	Pneumonia
P-47	1	2	1	0	2	1	0	0	0	0	1	0	1	Pneumonia
P-48	0	2	1	1	0	1	1	0	0	1	1	1	1	Pneumonia
P-49	2	0	1	0	0	0	1	1	0	1	0	0	1	Pneumonia
P-50	1	0	0	1	0	1	1	0	0	0	0	1	0	Pneumonia
P-51	1	2	0	1	2	1	1	1	2	1	2	1	2	Pneumonia
P-52	1	1	0	1	1	1	2	2	1	1	1	1	1	Pneumonia
P-53	1	1	0	0	2	0	1	2	1	1	2	2	2	Pneumonia
P-54	1	1	0	1	2	1	2	2	1	1	1	2	2	Pneumonia
P-55	1	1	0	0	2	1	2	1	1	1	1	1	1	Pneumonia
P-56	1	1	0	1	2	1	2	2	2	2	2	1	2	Pneumonia
P-57	1	1	0	0	2	0	1	2	2	2	2	2	2	Pneumonia
P-58	1	1	0	0	2	1	1	1	2	2	2	2	2	Pneumonia
P-59	1	1	0	0	2	0	2	2	1	2	2	1	2	Pneumonia
P-60	1	1	0	0	2	1	2	1	2	2	1	2	2	Pneumonia

**Table 4:** Performance comparison of the proposed work with the existing methods.

Parameters Parameters	CNN	Transfer Learning	Fine Tuning	RF	NB	SVM	SVM	SVM	SVM
						(Sigmoid	(Linear	(rbf	(Polynomial
						Kernel)	Kernel)	Kernel)	Kernel)
					Accuracy				
					analysis				
Proposed	95.7	96.3	97.0	0.99	89	13	51	94	94
Ref. [52]	90	80	88	89	89	91	90	83	85
Ref. [53]	85	92	91	91	90	91	91	90	91
Ref. [57]	94	74	76	78	72	73	81	83	85
Ref. [59]	84	82	81	88	91	92	90	91	96
Ref. [60]	90	85	81	91	91	93	90	91	92
					Precision				
					analysis				
Proposed	0.96	0.97	0.99	0.88	1.00	0.31	0.34	1.00	0.96
Ref. [52]	0.83	0.91	0.89	0.84	0.85	0.86	0.88	0.91	0.88
Ref. [53]	0.91	0.94	0.92	0.95	0.92	0.92	0.94	0.97	0.98
Ref. [57]	0.96	0.94	0.95	0.97	0.95	098	0.97	0.98	0.97
Ref. [59]	0.88	0.87	0.86	0.83	0.91	.96	0.97	0.96	0.97
Ref. [60]	0.88	0.87	0.86	0.83	0.91	.96	0.97	0.96	0.97
					Recall				
					analysis				
Proposed	0.96	0.99	1.0	0.95	0.79	0.14	0.86	0.91	0.84
Ref. [52]	0.88	0.91	0.92	0.94	0.90	0.93	0.94	0.93	0.91
Ref. [53]	0.91	0.93	0.90	0.89	0.97	0.93	0.94	0.91	0.90
Ref. [57]	0.90	0.91	0.89	0.88	0.95	0.91	0.92	0.90	0.91
Ref. [59]	0.96	0.90	0.91	0.90	0.91	0.95	0.94	0.91	0.95
Ref. [60]	0.90	0.91	0.93	0.95	0.95	0.91	0.91	0.90	0.90
					F1-score				
					analysis				
Proposed	0.97	0.98	0.99	0.95	0.88	0.21	0.44	0.93	90
Ref. [52]	0.85	0.91	0.80	0.87	0.84	0.96	0.92	0.93	0.91
Ref. [53]	0.91	0.91	0.82	0.91	0.92	0.90	0.97	0.95	0.93
Ref. [57]	0.95	0.96	0.91	0.90	0.90	0.95	0.94	0.91	0.90
Ref. [59]	0.90	0.89	0.91	0.92	0.91	0.90	0.94	0.95	0.98
Ref. [60]	0.90	0.89	0.91	0.92	0.91	0.90	0.94	0.95	0.98

**Table 5:** K-fold analysis.

Parameters	CNN	Transfer Learning	Fine Tuning	RF	NB	SVM	SVM	SVM	SVM
						(Sigmoid	(Linear	(rbf	(Polynomial
						Kernel)	Kernel)	Kernel)	Kernel)
					Accuracy				
					analysis				
K = 25	95.7	96.3	97	92	88	12	50	90	91
K = 30	94.0	94.4	95	85	79	21	55	84	91
K = 35	93.9	94.6	96	95	89	12	50	94	95
K = 40	93.5	93.9	94.5	96	90	12	50	95	95
					Precision				
					analysis				
K = 25	0.96	0.97	0.99	0.93	1.00	0.31	0.33	1.00	1.00
K = 30	0.93	0.95	0.97	0.81	1.00	0.32	0.33	1.00	1.00
K = 35	0.92	0.94	0.95	0.94	0.98	0.31	0.32	0.97	0.97
K = 40	0.91	0.	0.93	0.92	1.00	0.32	0.32	1.00	1.00
					Recall				
					analysis				
K = 25	0.96	0.99	1.0	0.91	0.79	0.16	1.00	0.90	0.92
K = 30	0.95	0.96	0.97	0.81	0.62	0.33	1.00	0.80	0.91
K = 35	0.95	0.97	0.97	0.92	0.78	0.14	1.00	0.91	0.92
K = 40	0.94	0.95	0.96	0.96	0.82	0.13	1.00	0.92	0.91
					F1-score				
					analysis				
K = 25	0.97	0.98	0.99	0.91	0.86	0.21	0.46	0.93	0.92
K = 30	0.96	0.97	0.98	0.82	0.76	0.32	0.51	0.89	0.96
K = 35	0.95	0.96	0.97	0.92	0.90	0.21	0.51	0.94	0.91
K = 40	0.95	0.97	0.97	0.94	0.83	0.20	0.50	0.93	0.96

## Data Availability

Dataset used in this study is available on individual request.
